# Discomfort Avoidance and Desire for Normality Are Decision‐Making Drivers of Footwear Choice in Underserved Communities at Risk of Diabetic Foot Ulcer

**DOI:** 10.1002/jfa2.70186

**Published:** 2026-07-12

**Authors:** Krithika Anil, Neeraj Sharma, Sonal Patel, Jess Bailey, Catherine Smith, Sally Abey, Kayce Allcock, Richard Collings, Joanne Paton

**Affiliations:** ^1^ School of Health Professions, Faculty of Health University of Plymouth Plymouth UK; ^2^ Brain Research and Imaging Centre University of Plymouth Plymouth UK; ^3^ Central and North West London NHS Foundation Trust London UK; ^4^ Livewell Southwest Plymouth UK; ^5^ Torbay and South Devon NHS Foundation Trust Torbay UK

**Keywords:** decision‐making, diabetes, diabetic‐related foot ulceration, footwear, interview

## Abstract

**Background:**

Diabetes‐related foot ulceration (DFU) is a serious complication that can lead to lower limb amputation or death. Custom‐made footwear is a key preventative strategy, yet adherence to use is often low. The factors influencing decision‐making for footwear use remain unclear, particularly among underserved groups such as people from low socioeconomic and South Asian backgrounds. This study aimed to identify the psychological mechanisms underlying footwear choice and adherence in people with diabetes from low socioeconomic and South Asian populations.

**Methods:**

This study used a qualitative approach conducted in two parts. Part 1 explored footwear‐related experiences using one‐to‐one interviews informed by the COM‐B model (which states that there are 3 components to Behaviour: Capability, Opportunity, Motivation). Findings from Part 1 informed Part 2, a ‘think aloud’ qualitative study examining reasoning strategies behind footwear choices. Participants were adults living with diabetes at moderate to high risk of foot ulceration, with or without prior foot ulcers or experience of therapeutic footwear. Those with active foot ulceration were excluded. Interview data were analysed using thematic analysis.

**Results:**

Eleven UK‐based participants completed Part 1 (7 male, 4 female; median age 64; South Asian = 7; low socioeconomic status = 6). Seventeen themes were identified and categorised within the COM‐B framework to inform Part 2. Fourteen participants completed Part 2 (10 male, 5 female; median age 67; South Asian = 5; low socioeconomic status = 8). Two key reasoning mechanisms emerged: (1) Discomfort Avoidance Prioritised Over Clinical Advice, reflecting a tendency to prioritise comfort over medical recommendations, and (2) Desire for Normality, describing a preference for familiar or ‘normal’ footwear.

**Conclusion:**

Footwear decision‐making among people living with diabetes seems to be informed by reasoning based on discomfort avoidance and normalcy. These findings highlight the need for a person‐centred approach to footwear advice that may bridge the gap between footwear adherence and patients' reasoning mechanisms. These mechanisms must be confirmed in a replication study or in an appropriately large quantitative study.

## 1 Introduction

Diabetes‐related foot ulceration (DFU) is a serious complication that affects 19%–34% of people with diabetes [1], and may result in complications leading to lower limb amputation or death [1]. Prevention is therefore key to diabetic foot management [2]. One such preventative strategy is the use of custom‐made footwear, which provides appropriate fit and support, protects against trauma, and reduces elevated plantar pressures associated with DFU [3].

However, professional‐recommended footwear adherence (wear time during weight‐bearing activities) is low in people living with diabetes, averaging at 63% [4]. Reasons for low adherence is unclear; Jarl et al. [4] found no predictors of footwear adherence but observed lower adherence at home compared to away from home. Keukenkamp et al. [5] found that custom‐made footwear increased adherence indoors, raising adherence from 65% to 83% after 12 months. They suggested that participants found this footwear more comfortable to wear indoors and therefore more motivated to wear footwear indoors. These results indicate that adherence is driven by psychological factors related to comfort and motivation.

Paton et al. [6] found that patients prefer footwear perceived as easy and safe to wear, and that adherence may be influenced by alignment with their self‐image rather than perceived foot health [7]. This finding suggest that footwear adherence is influenced by complex psychological processes beyond general motivation or health awareness.

This is particularly important to consider for hard‐to‐reach populations who are often under‐represented in research. As a result, these populations may be overlooked or insufficiently supported by standard healthcare approaches and current understanding of these complex psychological processes. We held a research engagement forum that included podiatrists and orthotists (*n* = 4) with experience of managing patients living with diabetes to identify priorities that inform future research in the topic of footwear and DFU. A key priority was the lack of evidence exploring the perspectives of individuals from low socioeconomic and South Asian backgrounds, perceived as two of the largest underserved groups in the UK. This aligns with UK census data showing that ‘Asian, Asian British or Asian Welsh’ individuals (including those of South Asian heritage) comprised 9.3% (5.5 million) of the overall population in 2021 [8], alongside an average of 56% of the UK population who were income deprived in 2025 [9]. People from low socioeconomic backgrounds may face financial barriers, while South Asian communities may experience linguistic barriers and culturally specific beliefs about health and treatment. In the UK, bespoke therapeutic footwear for the prevention and healing of DFU's is predominantly prescribed by orthotists and is provided free at the point of issue through the National Health Service (NHS), although footwear advice may be delivered by any appropriately skilled healthcare professional, including podiatrists. Yet, indirect financial costs (e.g., travel, time off work, etc.) and broader socioeconomic constraints may still influence engagement with services and adherence to footwear recommendations. These contexts may reduce uptake and sustained use of professional‐recommended footwear.

However, currently no study has yet examined adherence or related influencing factors in the South Asian populations. Ng et al.'s [10] recent systematic review on DFU treatment adherence found limited research addressing socioeconomic factors, and no significant effects identified. This is unexpected as other studies have found that socioeconomic factors (mainly functional social support and being employed) significantly influenced adherence to general medical treatments and management [11, 12, 13]. Ng et al. [10] concluded that the impact of socioeconomic factors may depend on the type of treatment, and that treatments involving self‐management behaviours are especially prone to being influenced by socioeconomic factors because they rely on personal and financial resources.

Despite emerging recognition that footwear adherence is psychologically and contextually determined, there is a lack of theory‐informed research explaining how people living with diabetes make footwear decisions, especially in low socioeconomic and South Asian populations. Understanding these mechanisms is essential for developing foot care that is both effective and acceptable, especially as patients may reject advice that conflicts with their lived experiences [14].

This study aimed to identify the psychological mechanisms underlying footwear decisions and adherence in people with diabetes from low socioeconomic and South Asian populations. The objectives were (1) to explore footwear‐related perceptions and experiences of people living with diabetes from low socioeconomic and South Asian populations that influences their footwear choices and decision‐making, and (2) to understand the reasoning strategies used to make footwear choices based on the outcomes from the first objective.

## 2 Methods

### 2.1 Study Design

This study was conducted in two parts: (1) the first part explored the contextual experiences of people living with diabetes and how that informed their footwear choice, and (2) the second part identified the reasoning strategies that drove behaviour in people with diabetes and their footwear choice.

#### 2.1.1 Part 1

Part 1 employed a one‐to‐one, cross‐sectional interview design that used a semi‐structured interview schedule. The interview schedule design was informed by patient and public involvement (PPIE) activities (further details below), and underpinned by the COM‐B model [15]. The COM‐B model stands for Capability, Opportunity, Motivation, and Behaviour, and has been widely used to explore influences of health behaviours. Capability describes an individual's psychological and physical ability to engage in certain behaviours. Opportunity describes social and physical factors that make a behaviour possible. Motivation describes the automatic and reflective cognitive processes that influence behaviour outcomes. Following consent, an audio‐recorded, in‐depth, semi‐structured interview lasting up to 1 h was conducted at the participants' preferred setting (NHS setting, participant's home, or telephone). This study used the consolidated criteria for reporting qualitative research (COREQ) to ensure transparency and quality [16], see the attached ‘Supporting Information S1—COREQ’ that also includes information for Part 2.

#### 2.1.2 Part 2

Using the outcomes from Part 1, scenarios were developed for a ‘think aloud’ qualitative study [17, 18]. Data collection and analysis procedures were conducted as described in Part 1: following informed consent, participants completed an audio‐recorded session lasting up to 1 h at their preferred setting (NHS setting, participant's home, or telephone).

### 2.2 Patient and Public Involvement

We organised a public forum, where people living with diabetes and at risk of foot ulceration (*n* = 6; all attendees had a South Asian heritage, two were from a low socio‐economic background) were invited to discuss our interview schedule. Attendees were identified and invited by their usual care team at the Central and North West London NHS Foundation Trust (UK), and through an advertising poster that was displayed in the patient waiting area of the clinic. If attendees wanted to find out more information, they were able to contact a member of the research team whose details were provided by the usual care team and via the poster. Attendees were introduced to the research topic by K.A. with a 5‐min presentation using plain English. S.P. and J.B. facilitated the discussion that followed, guided by open‐ended questions asking about attendee's thoughts on the importance, relevance, and phrasing of potential questions for the interview schedule. K.A. took observational notes. The outputs from this session informed Part 1's interview schedule.

### 2.3 Interview Schedule

#### 2.3.1 Part 1

The interview schedule for Part 1 was developed using previous literature, patient and public involvement, and the COM‐B model. After three interviews, interviewers gave feedback that it was difficult to gain in‐depth answers from those with English as a second language. The interview schedule was refined to address this issue by including pictures of footwear. The initial interview schedule and the refined interview schedule can be seen in the attached ‘Supporting Information S2—Interview Schedules P1’.

#### 2.3.2 Part 2

Initial scenario content was derived from Part 1 themes, which ensured each scenario represented a realistic footwear‐related decision point from which we could explore participants' footwear use rationale. Draft scenarios were refined through iterative pilot‐testing with independent lay people and expert review by the research team. The final scenarios used clear, accessible language and were designed to enable participants to articulate their cognitive processes during the think‐aloud task. The scenarios can be seen in the attached ‘Supporting Information S3—Interview Schedule Part 2’. We have briefly described the interview schedule here: this interview schedule included six footwear‐related scenarios reflecting decision‐making contexts identified from Part 1 interviews. Participants were instructed to verbalise their thoughts in real time while responding to each scenario, with minimal prompting. A brief practice task related to ordering a drink at a coffee shop was conducted to familiarise participants with the think‐aloud process. The term ‘shoes’ was used instead of ‘footwear’ as participants from Part 1 used ‘shoes’ and so this was deemed to reflect participants' language better.

The scenarios described the following: (1) seeking footwear advice following a podiatry consultation, (2) thoughts about accepting and being fitted for therapeutic shoes, (3) immediate and longer‐term use of therapeutic shoes, (4) purchasing new shoes when existing shoes wear out, (5) footwear choices for special social events, and (6) footwear use in indoor settings. The interview concluded with reflective questions exploring key influences on decision‐making, perceived challenges, and any other thoughts they may have had.

### 2.4 Participant Recruitment

Participant recruitment was the same for parts 1 and 2. Eligibility criteria were adults living with diabetes and at moderate to high risk of foot ulceration (IWGDF [19]), and were recruited from two NHS community podiatry sites: Central and North West London NHS Foundation Trust (London, UK) and Livewell Southwest (Plymouth, UK). Foot health information documented as part of usual clinical records were used to stratify participants into foot risk status categories.

Purposive sampling was used to recruit participants from underserved background, that is, those from a South Asian background and from a low socioeconomic background. South Asian countries included: Afghanistan, Bangladesh, Bhutan, India, the Maldives, Nepal, Pakistan and Ski Lanka. Low socioeconomic background included: occupation was semi‐skilled, unskilled, or unemployed; highest level of education was at Level 1 or 2 (GCSE or equivalent high school qualification). Exclusion criteria were active diabetic foot ulceration or low risk of diabetic foot ulceration, people residing in nursing or care homes, inability to communicate in any of the following languages: English, Gujarati, Hindi, Urdu, or Punjabi at a level considered appropriate to conduct an interview unless accompanied by a relative or friend willing to translate.

Potential participants were identified by their usual care team either through routine NHS footcare appointments or via postal letters. Letters were posted to people identified from the podiatry caseload screened as diagnosed with diabetes and living in the most deprived postcodes of London and Plymouth (Index of Multiple Deprivation 2019 [20]). Identified individuals received an information sheet with the opportunity to ask questions to the research team.

Recruitment was difficult as people from underserved populations prove more difficult to reach compared to the general population [21, 22]. Therefore, we invited participants from Part 1 to participate in Part 2. Three participants were involved in both Part 1 and Part 2 interview studies. The analysis section of this paper details how we assessed potential influence these participants may have had on the analysis outcomes.

### 2.5 Study Procedure

The study procedure was the same for parts 1 and 2. Potential participants who were interested in taking part in the study were scheduled an interview time at their convenience either over the phone or in‐person, where they provided informed consent prior to being interviewed. The interviews were conducted by J.P., S.P., J.B., C.S., and N.S. J.P. was an experienced qualitative researcher. N.S. received formal and informal interview training. C.S., J.B., and S.P. have interview experience, and received informal training. To ensure consistency, all five interviewers received training and opportunities to practice using mock interviews using the interview guide prior to data collection for both parts 1 and 2. Regular team meetings were held throughout the data collection period to discuss and reflect on the interview process and emerging findings.

The interviews for both parts lasted between 10 and 45 min, and were audio recorded. The shorter interview length times reflects differences in some participants' communication styles and level of elaboration. In Part 1 interviews, the interviewers used prespecified prompts and spontaneous prompts to elicit more detailed responses. However, these participants showed discomfort or frustration at the prompts as they believed they had answered the questions sufficiently. The interviewers did not prompt further to maintain rapport and avoid distressing the participants. This only occurred with participants whose English was a second language, which may have impacted the responses from these participants and explain their discomfort at prompts for further elaboration. In Part 2, prompts and clarifications from the interviewer were discouraged by the think‐aloud approach.

### 2.6 Analysis

#### 2.6.1 Part 1

Interview audio recordings were transcribed verbatim using the Microsoft Word dictate function and checked by N.S. for accuracy. Transcriptions were analysed by K.A. in NVivo 14 using inductive thematic analysis to identify initial codes. These codes were then mapped onto the COM‐B domains, which served as the main analysis framework. Thematic analysis was carried out iteratively. The iterative process involved multiple rounds of coding and theme refinement, with regular discussions within the research team to address inconsistencies and refine the themes. Additionally, final themes were analysed for indicative associations with ethnicity and socioeconomic status using a framework matrix that charted participant demographics of ethnicity and socioeconomic status against the final themes. Data were narratively summarised within the matrix by theme and demographic to examine potential patterns of association.

#### 2.6.2 Part 2

Analysis for Part 2 followed the same inductive approach as Part 1 but without the mapping to the COM‐B domain; additional analytic steps were used to capture participants' reasoning. Transcripts were examined for explicit indicators of reasoning: initial reactions or first‐mentioned ideas, causal statements (e.g., use of ‘because’, ‘so’, ‘therefore’, ‘if… then’), justifications, comparisons, and references to personal rules. Final reasoning mechanisms were analysed for indicative associations with ethnicity and socioeconomic status using the same framework approach used in Part 1. To assess any potential influence of the three participants involved in both Part 1 and Part 2 interview studies, analyses were performed with and without their data. The results showed no meaningful differences.

## 3 Results

### 3.1 Part 1

#### 3.1.1 Participant Demographics

Eleven participants were interviewed (7 male, 4 female; median age 64 [41–83]). Eight participants were classified as high risk for DFU, while the remaining three participants were classified as at least moderate risk. Although the clinical records did not contain sufficient information to definitively classify these participants as either high or moderate risk, there was adequate information to support their classification as moderate risk. Three participants were White British, one participant was East African, and seven participants were South Asian. Six participants were from a low socio‐economic background. Four potential participants declined to take part. Only two individuals gave reasons (receiving palliative care) for declining to participate.

#### 3.1.2 Themes

Seventeen themes were generated according to the COM‐B model. Table 1 presents an overview of these themes alongside illustrative quotes.

**TABLE 1:** Overview of Part 1 themes categorised according to the COM‐B model.

Domain	Sub‐domain	Themes	Illustrative quote
Capability	Physical	Foot health symptoms make finding the right footwear difficult	‘Just [choosing shoes is] really difficult… because [shoes] are not really made for the feet that we have’—P7
		Once found, the right footwear helps foot health symptoms and walking	‘That's why these shoes are alright, because so far I haven't had any [walking] problems with these shoes… I'll have to get another pair exactly the same’—P1
	Psychological	Footwear advice perspectives	‘I would like [the podiatrist] to recommend a shoe to me which has been, well, [a] shoe that's suitable for my need at the time’—P10
		Perceived purpose of therapeutic footwear	‘You walk a lot better [with therapeutic footwear]. With them I can walk pretty good’—P1
Opportunity	Physical	Difficulty accessing healthcare	‘The buses are like every hour. Yeah, it's a bit awkward to get [to the podiatrist]’—P1
		Financial limitations restrict footwear choice	‘Well, it's just the fact that, you know, everything has a price and you might be talking to somebody, who generally needs one of these… customised shoes, and [the shoes are] twice the price of the shoes they're wearing… And the person themselves want to [buy them], but they can't afford it’—P10
		Challenges in finding ideal footwear when shopping	‘Yeah, it's not easy to find [ideal shoes in the] shop… If you found some fit, on the top is OK and but the back is too wide’—P2
	Social	Judgement from peers	‘I [like] a normal shoe, otherwise [therapeutic shoes are] going to make you embarrassed. You know what I mean?’—P7
		Relying on family for footwear support	‘Well, my Stepson influenced me, and he put me on to these New Balance 345 and I stuck with them’—P10
		Footwear choices for special occasions	‘I have a shoe for [special occasions] as well… Which are also GEOX, and they don't necessarily have to be very comfortable as long as they look smart’—P9
Motivation	Automatic	Indoor footwear habits and preferences	‘At home, I'm not wearing anything, sometimes socks’—P11
		Ordinary footwear preferences	‘Soft inside, so I like the soft shoes inside’—P6
		Therapeutic footwear preferences	‘[Therapeutic shoes] should be normal’—P7
		Impact of weather	‘Sometimes I wear Crocs in the summer [because] they're cooler’—P10
	Reflective	Avoid worsening foot health	‘If [the shoes are] suitable for me then I have to buy it… I don't want my feet to get spoiled’—P4
		Choosing footwear for ease of use in daily life	‘I've been watching out for these [shoes], ones you put in there, you don't have to [fix them]. You put your foot [in] and it goes right in there’—P5
		Fear of falling drives need for good footwear	‘In my life, only I'm worried about shoes. [The path] is slippery, I don't want to fall’—P11

#### 3.1.3 Capability (Physical)

##### 3.1.3.1 Foot Health Symptoms Makes Finding the Right Footwear Difficult

Participants face considerable difficulties when trying to find footwear that they believe meets the needs of their foot health. Participants reported various symptoms that hindered their footwear choice, such as swollen legs, that made wearing most shoes uncomfortable and impacting their walking.

I had surgery for my leg but [it] made it worse… it's very swollen. And they said to me that I need to get some certain shoes and insoles… And so it's getting difficult [to find shoes]… it's not really suitable.

(P4)

Some participants had mobility issues that prevented them from bending appropriately to put footwear on, which restricted them to finding footwear that were easy to grip and slip on.

You can't know where your foot's going, if you know what I mean. It is sometimes a bloody struggle.

(P5)

One participant stated that he had balance issues regardless of the shoes he wore, and opting for footwear that supported his walking.

Balance is a bit of a problem even with or without shoes because when you stand, you wobble. If I stand still and talk to someone. Like I'm always like going forward or back because, the balance is off. [Shoes] don't make no difference.

(P1)

##### 3.1.3.2 Once Found, the Right Footwear Helps Foot Health Symptoms and Walking

Once participants were able to find what they believe to be the right footwear that suited their foot health needs, they felt more confident and safer during their daily activities. The right footwear did not make their symptoms worse, highlighting participants' health worries when choosing footwear.

If I was to wear certain shoes which are not comfortable, I'm going to get like more swelling and I'm going to start again cracking and everything. So, I have to be a bit careful what I wear.

(P4)

I have confidence in the shoes that I wear at the moment… I feel as safe as I can be, and I'm feeling that they help my feet, with diabetes needs of keeping them healthy.

(P10)

Participants also reported being ‘comfortable’ that referred to footwear not being tight (‘Because it is loose and I can, like now, I'm moving my toes’—P3), gives more padding (‘It's like a little extra pad into the bottom of your foot as you're walking’—P5), helps in reducing pain (‘[Footwear] is important, you got to walk… it hurts up on my feet’—P1), and helping with walking (‘It made it lot easier for me to walk’—P1).

#### 3.1.4 Capability (Psychological)

##### 3.1.4.1 Footwear Advice Perspectives

Participants reported a range of sources from which they receive their footwear advice, which includes the internet, leaflets, their own judgement, the radio, shoe shop staff and their healthcare professionals.

Participants valued footwear advice from healthcare professionals, however there were inconsistencies in how this advice was processed by participants. Some participants could not remember the footwear advice they were provided, and others stated that their healthcare professional did not provide specific footwear advice.

[The healthcare professional] did [give me advice] at the time, but I can't remember exactly what word for word.

(P1)

People have asked do you have comfortable shoes? The answer is Yes. No one's ever looked [at my shoes], not in depth whatsoever on that ….

(P10)

Interestingly, one participant had no expectations to receive footwear advice from their podiatrist.

I didn't expect [the podiatrist] to talk to me about shoes. Frankly, I expected her to obviously look at my feet… Did I expect her to recommend a particular shoe? The answer is no.

(P10)

Participants voiced a clear trust in professional footwear advice and wanted more specific advice tailored to their needs.

I would prefer that [the healthcare professional] give me [the shoes], because they know what is better.

(P2)

Most participants preferred face‐to‐face advice directly from a foot health specialist, while one participant preferred a leaflet as English was not their first language and may have found it difficult to follow verbal advice. Interestingly, one participant did not want advice. They did not provide an explanation but stated that they simply buy shoes from their usual shop.

So, what happens is once I'm going through [the shop's website], if I see something I like, [I get it]. I do not try to educate myself, you know.

(P9)

##### 3.1.4.2 Perceived Purpose of Therapeutic Footwear

Participants generally believed that therapeutic footwear was related to their diabetes. One participant felt that this footwear was only needed during the healing phase after amputation of toes, indicating that therapeutic footwear was associated with specific medical purposes rather than for everyday wear.

Before I had diabetes, it was ok to wear any footwear… Because I feel warm and squeezed… I like to wear, you know, little bit wider [toe box]… But now when I had diabetes, I don't like that.

(P2)

You know, at the beginning I needed [the therapeutic shoes] because I couldn't walk properly… But now [the amputation] is all healed up. I'm just getting back to normal.

(P1)

Another participant also reported that socks in conjunction with therapeutic footwear as important.

That's really what I'm trying to say, that it's important, the socks, they go in tandem… Change of socks every day and they should be, they should be feel firm within the shoe.

(P10)

#### 3.1.5 Opportunity (Physical)

##### 3.1.5.1 Difficulty Accessing Healthcare

Accessing healthcare was a barrier to receiving foot healthcare and advice. Participants felt that the services provided by a podiatrist were being reduced, which made it more difficult to arrange a face‐to‐face appointment.

You… can't get through. One time I phoned [the clinic], you had to come up regularly [to see them] and then all of a sudden, they're all finished… Because they don't cut nails anymore.

(P5)

Participants also felt that accessing healthcare services generally was becoming more difficult as many UK health services only arrange appointments online. The questions on the online system can be overwhelming and confusing, prompting the participant to abandon seeking care altogether.

It's hard to book an appointment… When I call the GP, they ask you to book online, and the system online is hard, it is hard because a lot of questions. Some questions you didn't understand, [that's] a problem. [The questions are] not finishing… So I left.

(P2)

Travel to appointments also posed a challenge, particularly for those in rural areas or those with limited mobility and financial resources. Bus passes (free for those over 60 years old) made the travel more accessible, yet they still faced long wait times for infrequent foot health services and often weighed the cost of travel against the perceived necessity of the visit.

I just paid the bus when I got whenever, but it cost a lot of money… I've got a bus pass [now], but the buses are like every hour. Yeah, it's a bit awkward to get in there… [The clinic] keep telling me to keep my appointments because the podiatrist [helps] your feet and cuts your toenails [but I] miss them some… I'll go to most of them. So it's a way I don't have to go bankrupt.

(P1)

##### 3.1.5.2 Financial Limitations Restrict Footwear Choice

Financial limitations were a prominent barrier in obtaining ideal footwear. Participants reported being aware of the type of footwear needed to support their feet but were unable to afford it. Participants noted the trade‐off they were making when purchasing the more affordable footwear, where this footwear wore out quicker than the more expensive footwear. One participant relied on their family to purchase footwear for them due to their personal financial limitations.

The cheaper pairs are no good, you know. They wear out, they break, and they go uncomfortable, quite quick.

(P1)

I wanted to be comfortable. I want nice shoes, [which were] £95. My children bought [me them as a] Christmas present. If I am alone, I am not giving £95.

(P11)

Participants also reported that proper footwear use is costly. Cleaning socks every day or buying footwear recommended by their foot health professional was expensive.

Yeah I wear socks [every day]… I've got to pay for [the cleaning]… So you pay 3 pounds every wash… So that's more money spent on things.

(P1)

They been there to doctors many times and then they advised them [particular] shoes, so they went to shoe shop. [But] it is too expensive.

(P2)

Some participants stated that they would prefer multiple footwears for different situations, but their finances restricted them to having only one pair of shoes.

For my gym. You know, for running. It is hard to run with these. I do not have special [footwear for running]. I want something special to wear [during] the run.

(P7)

##### 3.1.5.3 Challenges in Finding Ideal Footwear

Participants frequently described difficulties in finding suitable footwear that met both foot health and personal needs. The footwear may fit correctly but the style was not preferred, or the footwear only fits in certain areas and not others. Most participants stated that no single footwear fits exactly right.

[Sometimes] you when you find the readymade [footwear] in the shop, but then you don't find the style? That is the problem.

(P2)

Obviously different brands, they have a different kind of shoes and all this, but in my opinion we need some kind of special shoes for the diabetes people.

(P7)

Participants generally compromised by wearing footwear that were ‘good enough’, despite their negative impact on their foot health such as discomfort after prolonged wear or the need for frequent self‐made adjustments. This often led to long‐term discomfort and foot problems when shoes were worn for extended periods of time.

It's just me adjusting with [my footwear], so it's not helping… And obviously for my condition it's just me having to compromise.

(P4)

This one this trainer… When I use this for long time, I feel issues with it.

(P2)

#### 3.1.6 Opportunity (Social)

##### 3.1.6.1 Judgement From Peers

Participants were reluctant to wear therapeutic footwear as it signalled their diabetes to their peers. The appearance of such footwear was perceived as visibly marking them as different and were perceived to draw unwanted attention or judgement. This perception often led to feelings of embarrassment and a preference for more discreet footwear.

I [like] a normal shoe, otherwise [it is] going to make you embarrassed. You know what I mean? And you're going to make you different than this other people.

(P7)

Participants also shared conversations with peers about the high cost of therapeutic footwear that became a shared frustration. These discussions reinforced the perception that such footwear were unaffordable for the average person.

I know one person, he went there in that shop and… He liked that one, it is really OK for him. Because he has an issue with his feet for a long time. And where he work, they give him a good job, but this is more than £500… I said no way, £500 for shoes!

(P2)

##### 3.1.6.2 Relying on Family for Footwear Support

Some participants relied on their family for advice and financial support for ideal footwear. This reliance often resulted from mobility issues or financial limitations, which made it difficult for participants to independently find suitable footwear. Participants received help and advice from their adult children, who either recommended footwear or bought footwear for them.

I must go to my daughters and ask to bring me [footwear], I can't go and that far, to the shop.

(P11)

##### 3.1.6.3 Footwear Choices for Special Occasions

While participants were generally mindful of their foot health, special occasions presented a conflict between foot health needs and social presentation. The majority of participants prioritised the style of their footwear over comfort and foot health for special occasions, such as weddings and parties. The need to follow social norms, avoid peer judgement, and feel confident in one's appearance often outweighed professional recommendations.

I have [footwear for special occasions] because I brought them from my country, but it's not very comfortable.

(P6)

When I went outside… I prefer to go with the things I have been told by my doctor… but when I have [special occasions], I like to wear my style.

(P2)

Few participants prioritised comfort and foot health over style for special occasions. They often framed their behaviour as a practical necessity rather than a choice due to financial limitations or past experiences of discomfort.

As long as [the footwear] is clean and comfortable [for the special occasion]. We just give them a good polish.

(P5)

#### 3.1.7 Motivation (Automatic)

##### 3.1.7.1 Indoor Footwear Habits and Preferences

The majority of participants had different footwear habits indoors compared to outdoors. Many had footwear specifically for indoor use, which included Crocs, sandals, slippers, and socks, while a few participants went barefoot. Participants were unable to verbalise a specific reason initially; when pressed, participants stated that it was part of their upbringing or that it was simply more comfortable. Those participants who wore different footwear rather than being barefoot indoors reported that they wanted to avoid dirt and protect their feet.

I don't know. First only, when [I was] small, I'm not wearing [on my feet indoors].

(P11)

Because I can't walk around barefoot. I've always worn slippers. So, it's just a habit. And also, I don't want my feet to get dirty and also to protect my feet.

(P4)

Minority of participants used the same footwear for outdoor and indoor use. One participant stated it was because they stay home often and therefore only needs one item of footwear, while another reported that their trainers were the only footwear they had and so had no choice to change into indoor footwear.

So you know he's at home a lot. He doesn't get dressed formally anymore in a suit very much… A running shoe or a soft sole shoe like this is ideal, and he doesn't have to think about it. He puts it on and it works.

(P10's partner who attended interview as support)

I just wear these shoes. That's all I've got. I haven’t got the slippers or flip flops or nothing

(P1)

##### 3.1.7.2 Ordinary Footwear Preferences

Comfortable was the main choice of description for ordinary footwear. When asked what comfortable means to the participants, answers included that footwear should be soft, light, not tight, sturdy, and supportive. One participant also reported that it should be easy to put on, such as having Velcro instead of laces or that the footwear should not get caught on their saree when walking. Perception of footwear was also important, where participants only bought footwear that were stylish or from a good‐quality brand.

A few participants stated that they choose footwear that is comfortable to them contrary to their health professional's advice. They were unable to verbalise their reasoning beyond that their preferred footwear was more comfortable than the recommended footwear from their health professional.

So, [the podiatrist] told me what kind of shoes to wear, but I normally just… I should wear trainers, but obviously I can't always wear trainers. Basically, just depends… Because I like to wear shoes and all that… Comfy, so yeah.

P4

Interestingly, one participant preferred the footwear given to them as a Christmas gift by their late son who at the time lived in a different country due to the COVID‐19 pandemic. The footwear had great sentimental value and brought comfort when wearing them. While uncommon, this reason is a strong and personal motivator to wear footwear that may not be recommended by a health professional.

[I have] one pair of shoes. I liked it, this one only, nice shoes, comfortable. [My son] send me… Suddenly the lockdown came, and his job is gone. He went back to [home country]. He went back, within one year, he died. Virus is going on… We can't travel… I never seen him. Now, I wear his shoes two three years… still there.

(P11)

##### 3.1.7.3 Therapeutic Footwear Preferences

Participants reported the same preferences for therapeutic footwear as for ordinary footwear. An additional preference was that therapeutic footwear should look like ordinary footwear to avoid signalling their diabetic status to peers.

[Therapeutic footwear] should be normal way. Otherwise, when you have… the different look shoes, I mean they they're going to find you [are] different… I mean, they should have shoes like any other shoes.

(P7)

##### 3.1.7.4 Impact of Weather

Participants preferred to have different footwear for different weather, such as open footwear for warm weather and closed footwear during the rain or cold weather. However, some participants currently wore the same footwear no matter the weather, and most did not verbalise their reason. One participant reported that they choose not to leave their home during rain and therefore did not need another item of footwear.

No, I don’t go [out] when it is raining outside. It's very difficult.

(P6)

#### 3.1.8 Motivation (Reflective)

##### 3.1.8.1 Avoid Worsening Foot Health

Interestingly, only one participant mentioned choosing footwear to avoid worsening their foot health for example, swollen feet or balance issues. Other participants focused on aspects such as comfort, cost, or style, or reported that they choose footwear to help alleviate their foot health symptoms, as shown under the ‘Capability (Physical)’ section above.

If [the shoes are] suitable for me then I have to buy it… I don't want my feet to get spoiled.

(P4)

##### 3.1.8.2 Choosing Footwear for Ease of Use in Daily Life

Participants choose footwear that were practical in their daily life, rather than as a health‐related priority. Various features of footwear were chosen that were perceived to be good for their daily life, which included comfort, alleviation of foot health symptoms, stylish, steadiness while walking, easy to put on and secure, and suitability for their daily activities.

I've stuck with [my current footwear] all the time because they've met my need. They were comfortable from the word go. They gave me a steadiness, which I was looking for, and the style that I was looking for, and I've not really varied from that type of shoe.

(P10)

Some participants did not verbalise a specific feature, but choose footwear based on trial and error until they found suitable footwear they liked, showcasing that their choices were made through trial‐and‐error.

I just [get footwear]… Trial and error, yeah.

(P1)

##### 3.1.8.3 Fear of Falling Drives Need for Good Footwear

One participant emphasised a need to have good‐quality footwear to address their feel of falling while walking. This awareness was heightened due to past experiences of falling at home, where their spouse cautioned that their fall may lead to their death if they were not careful.

I am getting only scared. I'm falling a lot back home first. My husband said, one day you fall and you will die. That is in my mind.

(P11)

##### 3.1.8.4 Examination of Ethnic Backgrounds and Socioeconomic Status

Themes were examined to identify potential indications of associations with participants' ethnic backgrounds and socioeconomic status. It is important to highlight that this study is qualitative in nature; therefore, any observed associations are indicative rather than conclusive.

Participants from low socioeconomic backgrounds frequently described barriers to accessing healthcare, including challenges related to transportation and organising healthcare appointments. This theme did not appear within participants from higher socioeconomic backgrounds. Financial limitations were also only associated with low socioeconomic backgrounds.

Footwear style and presentation for special occasions emerged mostly among South Asian participants. However, while comfort was prioritised for White British and East African participants, they mentioned that presentation was still important, and ensured to clean and polish their comfortable footwear prior to a special occasion.

The authors were particularly interested to examine any demographics associations related to indoor footwear habits based on initial assumptions that they may influence footwear choice. Interestingly, indoor footwear habits appeared to be influenced more by personal preference rather than by ethnic background or socioeconomic status. While most participants reported using different footwear indoors and outdoors, two participants used the same footwear in both settings and provided specific reasons for doing so. One participant was limited by finances and could only afford one item of footwear, while the other mostly stayed indoors and did not need to buy specific outdoor footwear. Neither participant verbalised a reason for wearing footwear indoors, as opposed to wearing socks or going barefoot.

### 3.2 Part 2

#### 3.2.1 Participant Demographics

Fourteen participants were interviewed (10 male, 4 female; median age 67 [41–85]). Nine participants were classified as high risk for DFU, while the remaining five participants were classified as at least moderate risk. Although the clinical records did not contain sufficient information to definitively classify these participants as either high or moderate risk, there was adequate information to support their classification as moderate risk. Seven participants were White British, one participant was White Hungarian, one participant was British Asian, and five participants were South Asian. Eight participants were from a low socio‐economic background.

#### 3.2.2 Reasoning Underlying Footwear Use Decision‐Making

Before detailing the identified reasoning mechanisms, it is important to highlight the concept of discomfort in participants' responses. Participants consistently elaborated on references to ‘comfort’ by describing efforts to avoid physical discomfort (Table 2), suggesting that ‘comfort’ explicitly meant discomfort avoidance. ‘Comfort’ is a common term in footwear literature, but it's meaning can be unclear. Specifying comfort as discomfort avoidance is therefore crucial for understanding footwear choice and adherence to therapeutic footwear among people living with diabetes. This concept is further considered in the Discussion section.

**TABLE 2:** Discomfort avoidance overriding clinical advice—Illustrative quotes.

Participant ID	Footwear comfort	Clinical advice
P3	‘I collect [the therapeutic shoes]… And then when I open the box, yeah, [I see] if it's fit me, it's good. I keep it for me. If it's not fit me, I contacted [my clinician], and I say that one's not fit me’	‘I like to get [footwear] advice because my diabetic now is high… Last time the [clinician]… they tell me you [have] high blood and you are diabetic. Is not like last year. And the lady, I talked to her, [she] give me safety’
P8	‘Comfort… I think [footwear is] sort of soft… I have to be careful [with] what I wear, you know, because if I get bruises and stuff like that and then there's going to be a problem’	‘[Clinical advice] is good, because, since recently… I I'm losing my senses in my feet. Not as good as it used to be. So, I'll take it seriously, the advice’

Two main reasoning mechanisms were identified: (1) Discomfort Avoidance Prioritised Over Clinical Advice and (2) Desire for Normality.

##### 3.2.2.1 Discomfort Avoidance Prioritised Over Clinical Advice

This mechanism describes the tendency for participants to prioritise the avoidance of footwear‐related discomfort as their main decision criterion, often overriding clinical advice from healthcare professionals. This was despite participants greatly valuing the footwear advice from healthcare professionals to prevent diabetic‐related foot complications. See Table 2 below for illustrative quotes.

While participants trusted clinicians as the authority on foot health, the actions they take prioritise their own daily experiences that are rooted in comfort. This seems to reflect a dichotomy where short‐term relief of discomfort takes precedence over long‐term health, suggesting that participants see themselves as the expert when it comes to managing their day‐to‐day activities and lifestyle.

This phenomenon is not a conscious rejection of professional recommendations but seems to be a tendency to self‐validate decisions through lived experience. The immediate relief of discomfort seemed more important to participants than the long‐term health benefits of following clinical guidance. See Table 3 below.

**TABLE 3:** Quotes illustrating self‐assessment of footwear.

Participant ID	Self‐assessing footwear
P2	‘Normally, if I'm not either working or going for a function… [the therapeutic shoes] are the normal ones [that I wear]… The walking shoes appear to be the most comfortable [when on walks] because they [are] made to take on the kind of streets I [walk]’
P5	‘My first thought [when receiving therapeutic shoes] would be, oh I guess, just to see and try them on and see if this improves my issues and circulation in my feet’

##### 3.2.2.2 Desire for Normality

This mechanism describes participants' instinctive preference for choosing familiar or ‘normal’ footwear and was observed in their tendency to revert to ‘normal’ shoes once current footwear wore out (therapeutic or recommended commercial footwear by clinician). This was observed even when participants were presented with a scenario to select one of four shoes that included a pair of free therapeutic shoes (Table 4).

**TABLE 4:** Desire for normality—Illustrative quotes.

Participant ID	Obtaining new shoes	Choosing a shoe
P1	‘Just for general kickabout shoes, I'm getting a pair of trainers. But my dress shoes, they will come from Clarks, because I go there’	‘The pair that feel supported when you try them on, but they are expensive… [From the orthotist], you could get that pair. They may not be stylish, but they're gonna be good for your feet. And then when you want, go and save your money up. Go and get the ones that are a bit expensive… If you can do it yourself, do it. If not, then you ask for the help until you can't do it yourself to improve that’
P9	‘For nearly ten years I'm wearing almost only Skechers shoes. Because these shoes gave me enough arch support’	‘Definitely the first option [the supportive but expensive shoes]. So that is supportive even if it is more expensive’

Participants consistently prioritised footwear that fit their immediate comfort needs (i.e., avoiding discomfort) or personal rules rather than their foot health (Table 5). For example, when choosing footwear for indoor settings, participants often prioritised comfort or adhered to house rules (e.g., no shoes indoors). Similarly, for special occasions such as weddings, participants chose footwear that either prioritised comfort or respected the formality of the event. Overall, the desire for ‘normal’ footwear reinforces the concept that participants prioritise short‐term relief of discomfort over long‐term health benefits.

**TABLE 5:** Quotes illustrating comfort needs and personal rules influencing footwear choices.

Participant ID	Indoor settings	Special occasions
P10	‘Depends on what I was going to do. I would put my shoes on if I was going out the garden. Or just walk around [in socks] on your feet if need be’	‘If it was a case of wedding or something like that. I'm not being rude, OK, you're in a church… [I would] put my dress shoes on for the half an hour, 45 min [of the ceremony]. If I'm sat there, you'll probably find that I've slipped them off and I'm sort of like, just got my toes inside them’
P11	‘I take my shoes off at home… In someone else's home, I take my shoes off and keep my socks on’	‘I wear trainers, I don't have any shame. If there is any function, I wear sari, I wear trainers only’

#### 3.2.3 Examination of Ethnic Backgrounds and Socioeconomic Status

Reasoning mechanisms were also examined to identify potential indications of associations with participants' ethnic backgrounds and socioeconomic status. No associations were found, indicating that these reasoning mechanisms operate independent of these demographics.

## 4 Discussion

Part 1 of this study found that participants experienced substantial physical barriers to appropriate footwear selection, including foot‐related symptoms (e.g., swelling, pain), mobility limitations, and balance issues. Finding suitable footwear improved perceived comfort, confidence, and safety. However, psychological capability was influenced by inconsistent or poorly recalled professional advice, with participants expressing a preference for clearer, tailored guidance from healthcare professionals. Opportunities to engage with optimal footwear were restricted by systemic and socioeconomic factors, including limited access to podiatry services, financial constraints, and difficulty sourcing acceptable footwear that met both clinical and personal preferences. It is notable that some participants spoke about the cost of therapeutic footwear as a constraint, as such footwear is available free from the NHS for both prevention and healing of DFUs. This may indicate that participants were referring to self‐purchased footwear, or it may reflect limited awareness of NHS provision. Future research should explore patients' understanding and experiences of accessing NHS footwear, and reasons for seeking alternatives outside NHS provision. Social influences, such as stigma associated with therapeutic footwear, seemed to shape footwear decisions. Footwear behaviours also seemed to be influenced by habitual practices and practicality rather than prevention of poor foot health. While some participants recognised the role of footwear in avoiding foot health deterioration, choices were more often guided by ease of use, perceived comfort, and trial‐and‐error strategies.

Part 2 of this study revealed two insights: first, the identification of discomfort avoidance prioritisation and a desire for normality seem to be the main reasoning mechanisms that influence footwear decisions; and second, these mechanisms seemed to operate independently of participants' ethnicity or socioeconomic background. This insight highlights the importance of understanding how footwear decision‐making is organised and conceptualised, which differs from typical interview approaches that primarily identify thematic factors that participants report as influencing their choices.

The findings from Part 1 showed that footwear use in people living with diabetes and at risk of DFU is multi‐faceted. The generated themes align with previous work demonstrating that this population balance their current individual needs and social norms with foot health symptoms when selecting footwear [7, 23]. Here, ‘symptoms’ is used from the participants' perspective to describe their own lived experience of their foot health, and not clinically defined symptoms. This may indicate that patient conceptualisations of foot health are constructed within their everyday lives, and may not necessary align with a clinician's understanding of a symptom. Interestingly, indoor footwear use appeared to reflect individual preference or financial constraint. This aligns with another interview study that concluded indoor footwear habits were a personal choice [7].

Part 2 findings revealed two reasoning mechanisms, discomfort avoidance prioritised over clinical advice and a desire for normality, that can be used to understand participants' footwear choice decisions. Participants consistently referred to avoiding discomfort when choosing footwear that were ‘comfortable’. This is an important clarification as it may lead to a more accurate operational definition of comfort in future research [24]. Discomfort is broadly defined as ‘a negative physical and/or emotional state, causing unpleasant feelings or sensations’ [25], which individuals seek to reduce or avoid. Therefore, footwear discomfort may have a relationship with footwear adherence. However, it is important to note that the definition of comfort used in previous research may not be the same as comfort as reported by participants in this study. It is therefore crucial to understand and clearly define the concept of discomfort in this field, including whether this concept refers to only physical sensations or whether it also includes human factors such as social discomfort or similar. Future research in the field of DFU prevention may further specify discomfort by examining its relationship to specific clinical symptoms and patient perspectives. Such work may improve the understanding of adherence to therapeutic footwear among people living with diabetes. A recent conceptual review [26] that explored the footwear needs of the general public states that comfort should be considered an outcome of meeting an individual's footwear needs rather than an inherent feature of the footwear itself. This indicates that footwear comfort is a dynamic concept that can change over time, and not a static feature. The review highlights how footwear comfort is highly individualised and context‐dependent, which further reinforces the need to define comfort specifically for people with diabetes and at risk of a DFU. Particularly, understanding the focus on avoiding discomfort and how it can change over time and according to patients' needs will be valuable.

The first mechanism revealed that clinical advice (related to both general and therapeutic footwear) was explicitly valued. Yet discomfort avoidance in the short‐term seemed to be prioritised over long‐term health risk mitigation. This pattern is a well‐documented behavioural principle, where people tend to discount long‐term health outcomes when weighed against immediate experiences [27, 28, 29]. Importantly, this behaviour should not be interpreted as resistance to clinical advice. Participants trusted clinicians for health knowledge but viewed themselves as the experts of their own feet in the context of their daily lives. The second mechanism suggests that the transition back to ‘normal’ footwear once current footwear was worn‐out was an automatic choice, even for participants who accepted therapeutic footwear. This indicates that participants may not understand the life‐long need for therapeutic footwear. Again, it is important to note that these behaviours reflect unconscious internal negotiations between health and identity that occur without explicit reflection, rather than an outright rejection of clinical advice.

Additionally, our findings indicate that some participants had misunderstood the intended purpose of therapeutic footwear, where they interpreted it in terms of immediate physical relief or specific medical needs rather than preventive foot care. This misunderstanding is important to address as it may influence adherence and the effectiveness of footwear in preventing DFUs. Enhancing patient education and tailored communication about the actual purpose of prescribed footwear could help align patient perceptions with clinical recommendations; however, the reasoning mechanisms identified in this study suggests that education alone may not be enough. As mentioned above, footwear comfort may be dynamic, resulting in the concept of footwear adherence also being dynamic. This is reflected in participants' value of short‐term relief of discomfort and the need to return to ‘normal’. Understanding how patients conceptualise the purpose of footwear may support therapeutic footwear adherence.

Collectively, these findings suggest that participants are balancing financial barriers, their lived experience of discomfort, and their need for normalcy when choosing footwear; clinical advice seems to operate within these domains rather than above them. This is supported by William et al.'s [30] conclusion that patient education sits within broader lived priorities such as patients' social support system or stage of life with diabetes, not above them. It also highlights that the potential cause of differences between low socio‐economic and South Asian participants are related to finance opportunities rather than reasoning mechanisms.

### 4.1 Clinical Implications and Future Research

The study's findings highlight the need for a nuanced approach to footwear use advice for people living with diabetes and at risk of DFU. Clinicians need to address not just the foot health needs of their patients but also the reasoning mechanisms and psychological and social factors that influence footwear choice decisions [31]. A more person‐centred approach that incorporates individual concepts of comfort, personal identity, and socioeconomic factors may improve adherence to therapeutic footwear recommendations. Explicitly considering socioeconomic factor is crucial as Part 1 findings reveal that financial limitations restrict the footwear available to individuals. This approach may build upon the IWGDF 2023 guidelines [3] that emphasises education, encouragement, and reminders for patients regarding foot health protective behaviours. However, these guidelines promote the concept that health knowledge modifies behaviour, but it does not consider how an individual's lived experience may contextualise that knowledge and its impact on behaviour.

As discomfort avoidance was identified as a potential key influence on footwear choices, future studies should examine the role of discomfort‐related clinical factors (e.g., sensory neuropathy, foot pain, or mobility impairment) and its impact on engagement and adherence with therapeutic footwear. It was also interesting to note that participants sometimes used clinical terms to describe their own experience, for example, the use of ‘symptoms’ as indicated by the theme ‘Foot health symptoms make finding the right footwear difficult’. This reflects a potential semantics misalignment between a patient and a clinician, which may impact treatment and management outcomes. Future research should also examine how these lay conceptualisations influence adherence and clinician‐patient communication.

Most importantly, the reasoning mechanisms must be confirmed in a replication study or a quantitative study with an appropriately large sample size. Throughout any future research that builds on these findings, a focus on understanding socioeconomic barriers is crucial. Even if patients understand the need for therapeutic footwear, such footwear may not be accessible if patients are financially limited.

### 4.2 Research Reflections

We faced several challenges around recruitment and communication. Reaching individuals from lower socioeconomic backgrounds was difficult as some were unfamiliar with what research participation involved or how it might benefit them, while others were wary of being contacted, even through legitimate clinical channels. Many preferred not to leave their homes due to financial constraints or fatigue.

During interviews, some participants gave very brief answers or had limited English proficiency (including those who had English as their first language). Although participants were offered interviews in their native language (e.g., in Hindi), participants preferred to speak in English.

Some interviewers (Patel, Bailey, and Smith) were also members of the participants' usual clinical care team. This decision was made because recruitment attempts highlighted that the target population was reluctant to engage with unfamiliar researchers. This reflected a broader distrust of strangers that contributed to their classification a ‘hard‐to‐reach’ population within the literature [32, 33, 34, 35]. Involving known clinicians facilitated recruitment and appeared to reassure participants that this was a legitimate research study. We acknowledge that this also introduced the potential for response bias, including social desirability effects or reluctance to express criticism of care. This possibility was discussed within the research team throughout data collection and analysis. To mitigate this, interviewers emphasised the voluntary nature of participation, explicitly clarified that participation (and non‐participation) would not affect clinical care, and encouraged honest reflection, including negative experiences. Ongoing reflective team discussions were used to examine how interviewer positioning may have shaped the data. Ultimately, the interviewers' clinical backgrounds helped to put participants at ease. Familiarity and pre‐existing trust often made the interviews feel more natural. It is important to note that this study would not have been possible without this process.

These experiences reinforce the need for research engagement approaches that prioritise relationship‐building, accessible communication, and sustained support particularly for individuals experiencing deprivation.

The wider research team included backgrounds in podiatry (Paton, Collins, Allcock, Abey), biomechanics (Sharma), and health psychology (Anil), which supported multidisciplinary research design and interpretation of findings. These varied disciplinary perspectives may have also influenced which aspects of participants' experiences were prioritised during interpretation, such as references to foot ulcer prevention, foot ulcer symptoms, footwear function, and behavioural factors underlying footwear choices. The research team therefore used ongoing team discussions throughout data collection and analysis to challenge each other's assumptions, compare interpretations, and consider alternative explanations that accurately reflects the data.

### 4.3 Strengths and Limitations

This research is the first to identify reasoning mechanisms for footwear choice using evidence‐based scenarios of lived experiences in people living with diabetes. The study designs were informed by current literature, real‐world clinical experience, and patient engagement exercises. There were also some limitations to this research. Firstly, there were multiple interviewers, which may have impacted the consistency of interviewing techniques and quality. Secondly, this study recruited individuals whose first language was not English. All interviews were conducted in English at the request of participants, and this may have impacted their ability to fully express their thoughts.

## 5 Conclusion

This paper reveals that footwear choice decision‐making among people living with diabetes is shaped less by ignorance or intentional dismissiveness of clinical advice and more by internalised reasoning based on discomfort avoidance and normalcy. These mechanisms go beyond demographics, except when socioeconomic disadvantage amplifies barriers to footwear. Health services should align recommendations with patients' lived experience, address comfort explicitly, and support individuals in integrating therapeutic footwear into their everyday identities and routines.

## Author Contributions

**Krithika Anil:** conceptualization, formal analysis, funding acquisition, methodology, validation, visualization, writing – original draft preparation, writing – review and editing. **Neeraj Sharma:** data curation, investigation, validation, project administration, writing – review and editing. **Sonal Patel:** investigation, writing – review and editing. **Jess Bailey:** investigation, writing – review and editing. **Catherine Smith:** investigation, writing – review and editing. **Sally Abey:** conceptualization, funding acquisition, writing – review and editing. **Kayce Allcock:** validation, writing – review and editing. **Richard Collings:** conceptualization, funding acquisition, validation, writing – review and editing. **Joanne Paton:** conceptualization, funding acquisition, investigation, supervision, methodology, validation, writing – review and editing.

## Funding

This study was part of a larger project that was funded by the Great Foundations charity for £116,298.

## Ethics Statement

Ethical approval was provided by the University of Plymouth's Faculty of Health Research Ethics and Integrity Committee (ID: 5404) and by the Health Research Authority's Research Ethics Committee (ID: 342944).

## Consent

All participants provided written, informed consent. Participants were notified that they may withdraw from the study at any time, even after providing informed consent and without reason.

## Conflicts of Interest

The authors declare no conflicts of interest.

## Supporting information


Supporting Information S1

Supporting Information S2

Supporting Information S3

## Data Availability

The raw and transcribed data is unavailable due to the sensitive healthcare nature of the content and to protect the participants' identities.
