# Supplementary material for: Discomfort Avoidance and Desire for Normality Are Decision‐Making Drivers of Footwear Choice in Underserved Communities at Risk of Diabetic Foot Ulcer
Source: J Foot Ankle Res. 2026 Jul 12;19(3):e70186. doi: 10.1002/jfa2.70186 (PMC13357689; doi:10.1002/jfa2.70186)
Supplement: Supplementary file 1 — Supporting Information S1 [file JFA2-19-e70186-s002.docx]

APPENDIX 1 - COREQ checklist

The Consolidated Criteria for Reporting Qualitative Studies (COREQ): 32-item checklist

| No. Item | Guide questions/description | Notes |
| --- | --- | --- |
| Domain 1: Research team and reﬂexivity | | |
| The research team  The multidisciplinary research team had expertise in podiatry and diabetes-related foot health (JP, CS, SA, RC, SP, JB, NS), diabetic foot ulcer prevention (JP, CS, RC), diabetic footwear (JP), health behaviour science (KA). | | |
|  | | |
| Personal Characteristics | | |
| 1. Inter  viewer/facilitator | Which author/s conducted the interview or focus group? | JP, CS, SP, JB, NS |
| 2. Credentials | What were the researcher’s  credentials? E.g. PhD, MD | PhD: JP, NS  BScs: SP, CS, JB |
| 3. Occupation | What was their occupation at the time of the study? | Assoc prof: JP; Research fellow: NS  Podiatrists: JB, SP, CS, JP |
| 4. Gender | Was the researcher male or female? | There were 4 female interviewers (SP, JB, JP, CS), and 1 male interviewer (NS). |
| 5. Experience and training | What experience or training did the researcher(s) have? | See section “Recruitment and Study Procedure”. |
| Relationship with participants | | |
| 6. Relationship established | Was a relationship established prior to study commencement? | The research team did not have any contact with participants prior to obtaining informed consent. Researchers had no professional or ongoing relationship with the participants. |
| 7. Participant knowledge of the interviewer | What did the participants know about the researcher? e.g. personal goals, reasons for doing the research | Participants were aware that this was a research project to explore their opinions on footwear and how they used their footwear in their daily lives. The research team explained that the goal of the research was to improve evidence-based foot health care for patients at risk of diabetic foot ulcers. |
| 8. Interviewer characteristics | What characteristics were reported about the inter viewer/facilitator?  e.g. Bias, assumptions, reasons and interests in the research topic | See section “Interview Schedule”. |
| Domain 2: Study design | | |
| Theoretical framework | | |
| 9. Methodological orientation and Theory | What methodological orientation was stated to underpin the study?  e.g. grounded theory, discourse analysis, ethnography, phenomenology, content analysis | The research team used the COM-B model, previous literature, and PPIE information to design and analyse their study. |

| 10. Sampling | How were participants selected? e.g. purposive, convenience, consecutive, snowball | We used a combination of purposive and convenience sampling. We attempted to reach maximum variation regarding age, gender, socioeconomic and cultural background for the patient participants. |
| --- | --- | --- |
| 11. Method of approach | How were participants approached?  e.g. face-to-face, telephone, mail, email | Patients were identified by their usual care team, who passed interested participants to the research team to continue the recruitment process. Participants were also approached using a mail drop, where the usual care team invited potential participants via a postal letter than instructed receivers to contact the research team if they were interested in the study. |
| 12. Sample size | How many participants were in the study? | 11 participants. |
| 13. Non-participation | How many people refused to participate or dropped out? Reasons? | See section “Participant Demographics”. |
| Setting | | |
| 14. Setting of data collection | Where was the data collected? e.g. home, clinic, workplace | Interviews were conducted over the phone and in-person at the University or in clinic, depending on the participant’s preference. |
| 15. Presence of non- participants | Was anyone else present besides the participants and researchers? | See section “Recruitment and Study Procedure”. |
| 16. Description of sample | What are the important characteristics of the sample? e.g. demographic data, date | See Introduction and section “Participant Demographics”. |
| Data collection | | |
| 17. Interview guide | Were questions, prompts, guides provided by the authors? Was it pilot tested? | See section “Interview Schedule” |
| 18. Repeat interviews | Were repeat inter views carried out? If yes, how many? | N/A |
| 19. Audio/visual recording | Did the research use audio or visual recording to collect the data? | All interviews were audio recorded with permission of participants. |
| 20. Field notes | Were ﬁeld notes made during and/or after the interview or focus group? | N/A |

| 21. Duration | What was the duration of the interviews or focus group? | 10-45 minutes. |
| --- | --- | --- |
| 22. Data saturation | Was data saturation discussed? | Yes, it was discussed. After collecting and analysing 2-3 interviews, team discussions were held that included reviewing the raw data and the resulting codes/themes. Once the team agreed that no new codes/themes were being generated, we were confident about our sample size. |
| 23. Transcripts returned | Were transcripts returned to participants for comment and/or correction? | No. |
| Domain 3: Analysis and findings | | |
| Data analysis | | |
| 24. Number of data coders | How many data coders coded the data? | See section “Analysis”. |
| 25. Description of the coding tree | Did authors provide a description of the coding tree? | N/A |
| 26. Derivation of themes | Were themes identified in advance or derived from the data? | Themes were derived from the data in an inductive manner, and categorized according to the COM-B model. |
| 27. Software | What software, if applicable, was used to manage the data? | Researchers used NVivo during the coding of all interviews. |
| 28. Participant checking | Did participants provide feedback on the findings? | There was no member check performed by the research team. |
| Reporting | | |
| 29. Quotations presented | Were participant quotations presented to illustrate the themes/ findings? Was each quotation identified? E.g. participant number | See section “Results”. |
| 30. Data and findings consistent | Was there consistency between the data presented and the findings? | All findings were derived from the data and all themes are supported by illustrative quotes. |
| 31. Clarity of major themes | Were major themes clearly presented in the findings? | Major themes were derived from the data and are clearly summarised and defined by a paragraph title. |
| 32. Clarity of minor themes | Is there a description of diverse cases or discussion of minor themes? | Themes on which there was a deviant opinion within the group or between the groups are discussed. Themes that are only described by one participant are also discussed in the results section of this study. |
