# Supplementary material for: Discomfort Avoidance and Desire for Normality Are Decision‐Making Drivers of Footwear Choice in Underserved Communities at Risk of Diabetic Foot Ulcer
Source: J Foot Ankle Res. 2026 Jul 12;19(3):e70186. doi: 10.1002/jfa2.70186 (PMC13357689; doi:10.1002/jfa2.70186)
Supplement: Supplementary file 2 — Supporting Information S2 [file JFA2-19-e70186-s001.docx]

**Initial Interview Schedule**

*Capability*

1. How would you describe the difference between ordinary footwear and specialised diabetic footwear?

*Prompts*:

- 1. What should the shoe look like?
  2. How should the shoe fit?
  3. What should the shoe be made of?
  4. How should the shoe feel?

1. How do you get your information and advice about what to wear on your feet and footcare?
2. If you have already been given some of this information can you share your experiences, if not do you have any thoughts on the best way to get given this information?

*Prompts*:

- 1. How would you prefer to get this information?
  2. What stops you from getting this information? (If applicable)

1. Can you tell me about how any foot-related issues may have made it difficult to wear certain types of footwear?

*Opportunity*

1. Think about the times you have shopped for shoes. Can you tell me about anything that helped you or made it easier to find appropriate footwear?

*Prompts*:

- 1. What made it difficult to find shoes?
  2. Was there anything else that influenced your shoe purchase?

1. In what ways do expectations at special occasions, for example at weddings or other occasions, impact your decisions when selecting footwear?
2. What do you wear on your feet when outside?

*Prompts*:

- 1. Tell me why you like wearing that outside?

1. What do you wear on your feet at home, if anything at all?

*Prompts*:

- 1. Tell me why you like wearing that (or not wearing anything) at home?
  2. What would it take for you to wear something on your feet at home to protect your feet?

*Motivation*

1. What do you consider as the most important footwear features that makes you decide to buy certain shoes?

*Prompts*:

- 1. For example, does it need to look or feel a certain way?
  2. Is there anything else that you may think about when deciding to buy shoes?

1. How does your current footwear help your foot problems?
2. How does your usual daily activities influence your footwear choice?

*Prompts*:

1. Are there any activities that you need special footwear for?
   - If yes, can you tell me about that special footwear and why you choose it?
   - If not, do you own several pairs of shoes? When do you wear them?
2. Do you have different footwear for different occasions?

*Prompts*:

1. If yes – Tell me about the different occasions and what footwear you choose for them.
2. If no – Do you think you need different footwear for different occasions? Why/why not?
3. Do you have different footwear for different seasons/weather?

*Prompts*:

1. If yes – Tell me about the different seasons/weather and what footwear would you choose.
2. If no – Do you think you need different footwear for different seasons/weather? Why/why not?

**Refined Interview Schedule**

*Capability*

1. How should a specialised diabetic footwear differ from ordinary footwear?

*Prompts*:

- 1. What should the shoe look like?
  2. How should the shoe fit?
  3. What should the shoe be made of?
  4. What should a specialised shoe be like for you to want to wear it?

1. Do you think of anything else about the design of specialist diabetic shoes
2. Style
3. Fit
4. 3)Upper material softness/stiffness

4) Fastening

5) Heel height

6) Sole tread

7) Sole/insole flexibility vs. rigidness

8) Sole/insole softness vs. hardness

1. How do you get your information and advice about what to wear on your feet and footcare?
2. If you have already been given some of this information can you share your experiences, if not do you have any thoughts on the best way to get given this information?

*Prompts*:

- 1. How would you prefer to get this information?
  2. What stops you from getting this information? (If applicable)

1. Can you tell me about how any foot-related issues may have made it difficult to wear certain types of footwear?

*Opportunity*

1. Think about the times you have shopped for shoes. Can you tell me about anything that helped you or made it easier to find appropriate footwear?

*Prompts*:

- 1. What made it difficult to find shoes?
  2. Was there anything else that influenced your shoe purchase?

1. In what ways do expectations at special occasions, for example at weddings or other occasions, impact your decisions when selecting footwear?
2. What do you wear on your feet when outside?

*Prompts*: Tell me why you like wearing that outside?

1. What do you wear on your feet at home, if anything at all?
2. What are your thoughts on the following shoe designs?

*Probe- function and cosmetics during different activities*


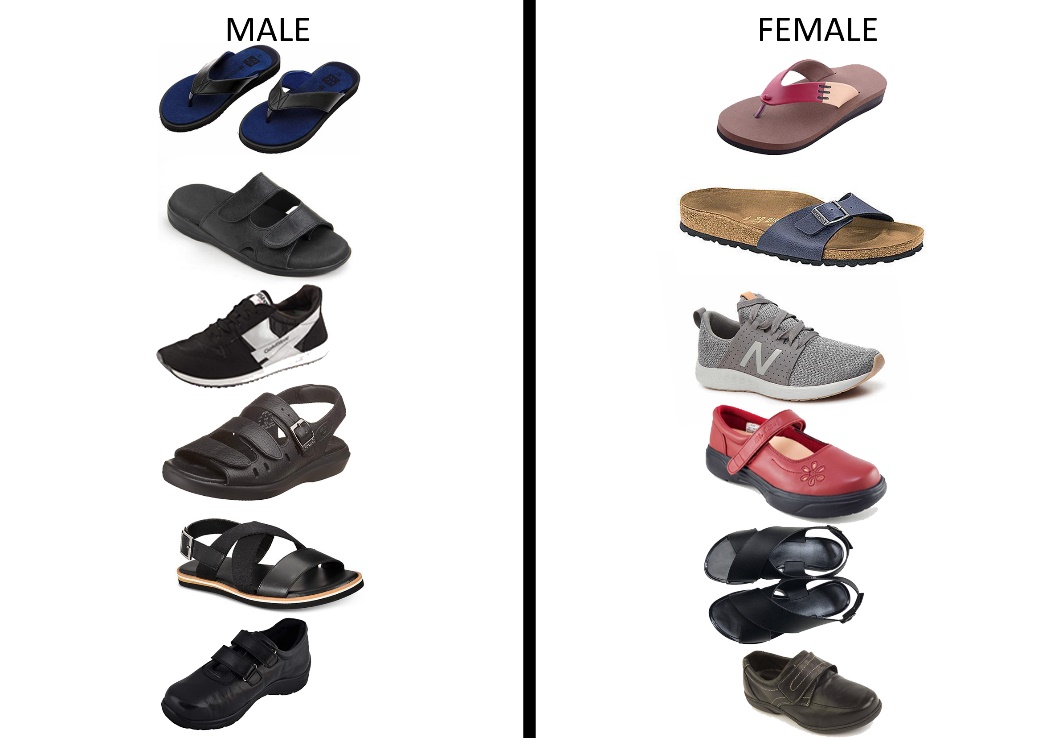


Which ones would you use? And why? Indoor / outdoor.

*Prompts*:

- 1. Tell me why you like wearing that (or not wearing anything) at home?
  2. What would it take for you to wear something on your feet at home to protect your feet?

*Motivation*

1. What do you consider as the most important footwear features that makes you decide to buy certain shoes?

*Prompts*:

- 1. For example, does it need to look or feel a certain way?
  2. Is there anything else that you may think about when deciding to buy shoes?

1. What do you consider most important and when choosing which shoes to wear. What is least important to you?

| Look | 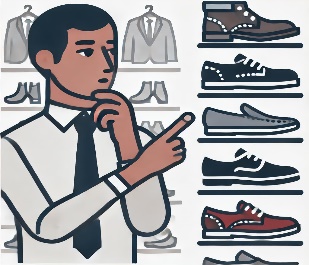 |
| --- | --- |
| Fit | 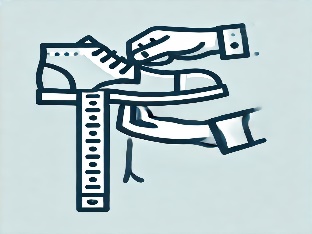 |
| Ease of Walking | 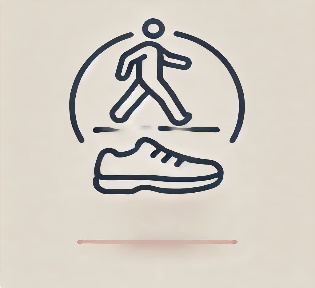 |
| Weight | 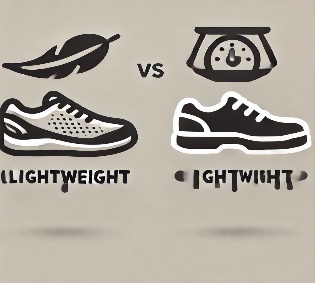 |
| Ease to put on | 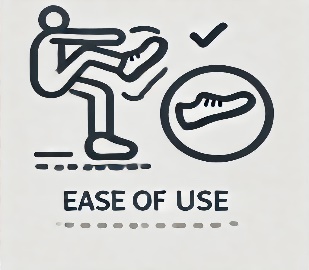 |
| Foot Protection | 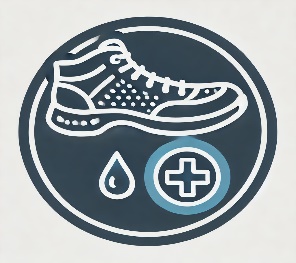 |

1. Is Comfort important? Can you tell me what footwear comfort means to you, when we talk about: (Show participant the picture of shoe and point towards specific features, you are inquiring about)


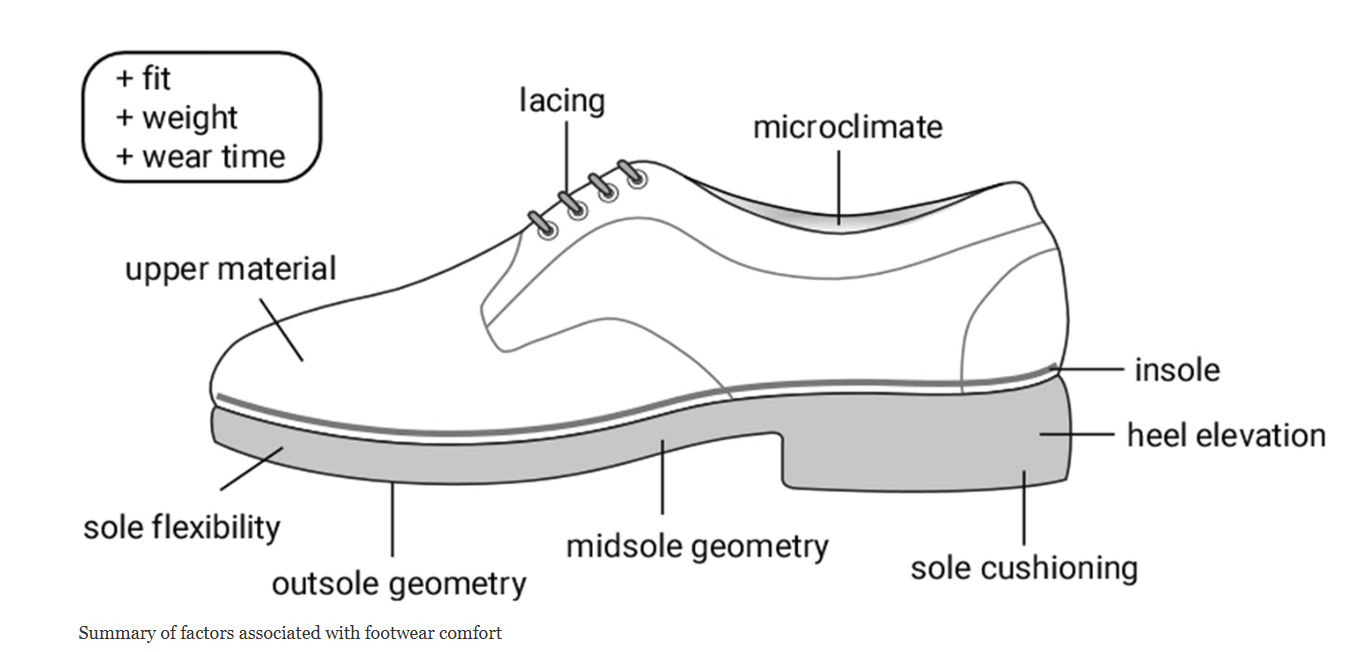
*Source: Menz et. al., 2021*

- **Fit:** What sort of shoe fit do you prefer (tight fit, spacious?)? Why?
- **Weight:** Does the weight of the shoe make any difference to your comfort? Why is that?
- **Softness:** Do you prefer a soft cushioned sole shoe or a firm supportive shoe? How does that help?
- **Flexibility**: How does the sole flexibility affect your comfort?
- **Temperature:**Do your feet most often feel hot or cold? – does that affect your choice of footwear? How?
- **Dryness:** Do you ever need your shoes to be waterproof? When? How often does that happen?
- **Arch Support:** Do you prefer a shoe with or without an arch support? Why?
- **Lacing System:** What fastening do you prefer? Do you like it done up tight or is it better loose? How does that help?
- **Heel Height:** What sort of heel do you find most comfortable? Very flat, a bit of a heel? Can you explain why that is?
- **Insoles:** Do you wear insoles? Tell me more about them?

1. How does your current footwear help your foot problems?
2. How does your usual daily activities influence your footwear choice?

Let’s play through a typical day so we can think about what you usually wear on your feet.

*Prompts*:

1. Are there any activities that you need special footwear for?
   - If yes, can you tell me about that special footwear and why you choose it?
   - If not, do you own several pairs of shoes? When do you wear them?
2. Do you have different footwear for different occasions?

*Prompts*:

1. If yes – Tell me about the different occasions and what footwear you choose for them.
2. If no – Do you think you need different footwear for different occasions? Why/why not?
3. Do you have different footwear for different seasons/weather?

*Prompts*:

1. If yes – Tell me about the different seasons/weather and what footwear would you choose.
2. If no – Do you think you need different footwear for different seasons/weather? Why/why not?
