# Supplementary material for: Discomfort Avoidance and Desire for Normality Are Decision‐Making Drivers of Footwear Choice in Underserved Communities at Risk of Diabetic Foot Ulcer
Source: J Foot Ankle Res. 2026 Jul 12;19(3):e70186. doi: 10.1002/jfa2.70186 (PMC13357689; doi:10.1002/jfa2.70186)
Supplement: Supplementary file 3 — Supporting Information S3 [file JFA2-19-e70186-s003.docx]

**Phase 2 Interview Schedule**

**Introduction Before Interview**

“I will read out six scenarios related to footwear choices. We want to know what you would do in each scenario and how you would make decisions about your footwear.

When you think about the scenarios, tell me your thoughts. I want you to talk out loud while you're making sense of the scenarios, so I can understand what you're thinking.

You do not need to explain your thoughts in detail, you just need to say what comes into your head.

Please tell me all your thoughts as you think them, even if you think they are unimportant. We would like to know everything that you may consider and any worries you have for each scenario. There are no right or wrong answers, no detail is too small. Everything you tell us will be helpful.”

**Practice Scenario**

“Let’s practice with one scenario so that you know what to expect. Please read the following and tell me your thoughts as they come to you”

“*You walk into a café on a sunny, warm day and want to order a drink. You look at the menu and see a selection of teas, coffees, hot chocolate, juice, and bottled fizzy drinks. Tell me what you think about when choosing a drink and why that was important for you to consider.*”

Post-scenario questions:

- How did the task feel for you?
- Would you like to try that again or do you feel comfortable enough to continue?

**Scenario 1: Getting Footwear Advice**


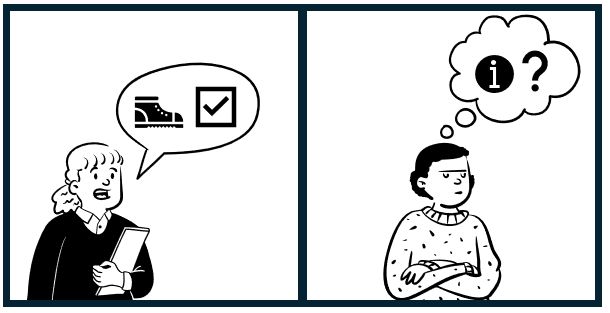


*Part 1*

“*Imagine you are at home following your podiatry appointment. Your podiatrist has advised you that wearing proper shoes is important to protect your feet.*

*As you think about the shoes you usually wear, tell me what you want to know about proper footwear and why this is important to you.”*

*Part 2*

*“There are lots of different ways you can get footwear advice. Tell me about how you would get more advice and why that works best for you.*”

**Scenario 2: Deciding on Therapeutic Shoes**


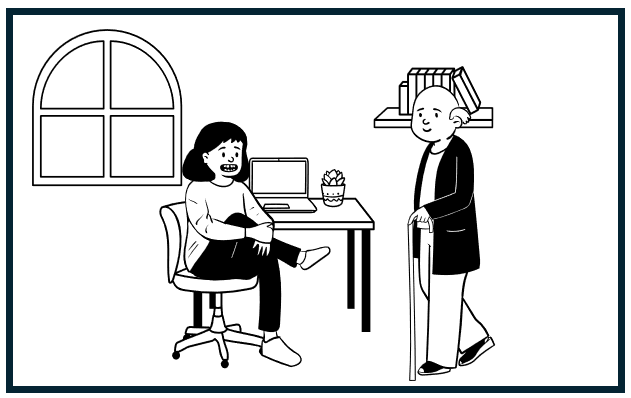


*Part 1*

"*Imagine you are at your foot clinic appointment with your podiatrist. They know about your diabetes and have checked your feet.*

*They say you “You have a loss of feeling in your feet and you are at risk of foot ulcers. I recommend you start wearing orthotic shoes to protect your feet”. What are about your first thoughts of this recommendation?”*

Further questions:

- “*Tell me about whether or not you would like to be referred for these therapeutic shoes*.”
- FOR INTERVIEWER: If the participant chooses not to accept these shoes, please move on to scenario 4.


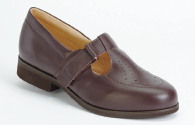

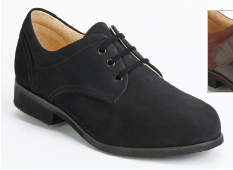

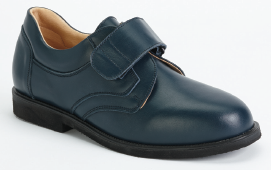

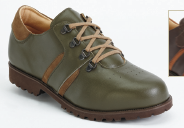

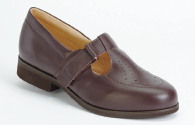

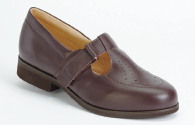


*Part 2*

*“After deciding to be referred for therapeutic shoes, you attend a fitting appointment a few weeks later with an orthotist.*

*The orthotist measures your feet to get the best fit for your shoes. They ask you about any areas that cause you pain or discomfort, your daily activities, and how long you usually spend on your feet.*

*At the end of the appointment, the orthotist tells you that your therapeutic shoes will be ready for a fitting in 4-6 weeks, and then they will be sent off to be finished and delivered for you to use.*

*They then ask “Do you have any questions or concerns about these therapeutic shoes?” Tell me how you would respond and what you want the orthotist to know.”*

**Scenario 3: Using the Therapeutic Shoes**

Please skip this scenario if the participant decided they would not accept the therapeutic shoes in the previous scenario.

*Part 1*


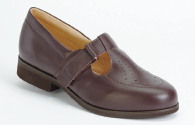

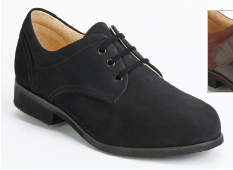

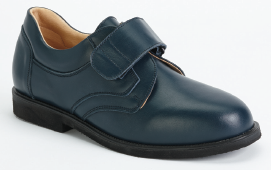

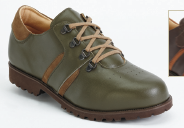

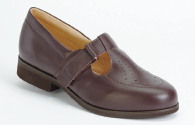

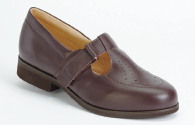


“*Imagine you have just come home with a box of your new therapeutic shoes. What are your first thoughts about the shoes and what is the first thing you do with them?*”

*Part 2*

“*You need to store your therapeutic shoes somewhere. Tell me where you decide to store them and why this location is useful for you.*”

*Part 3*

“*You go about your daily routine over the next few days. Tell me about the footwear you decide to wear and why this suits your daily routine*.”

*Part 4*

“*It is now 6 months since you’ve received your therapeutic shoes. Tell me about how you use the shoes now and why*.”

**Scenario 4: Choosing New Shoes**


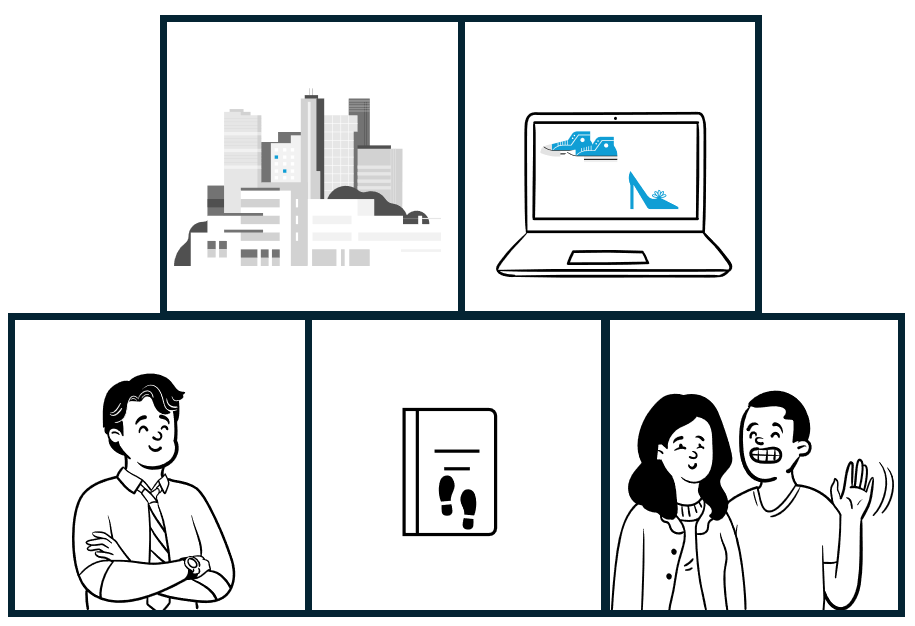


*Part 1*

“*You realise that your current shoes have worn out, and you need a new pair. You want something comfortable and that also follows the advice given by your healthcare professional. Where would you go to look for new shoes?*”

*Part 2*

*“After you explore your options, you find several different choices:*

- *A pair that feels supportive when you try them on, but they are expensive*
- *Another pair that is more affordable but doesn’t feel as supportive and may wear out quicker than the more expensive shoes*
- *There’s also a stylish pair in a colour that you like, but they feel tighter than you would prefer*
- *You also remember that you can go to your orthotist for a pair of therapeutic shoes at no cost*

*Tell me about how you would go about deciding the shoes you would buy.*”

**Scenario 5: Footwear For Special Events**


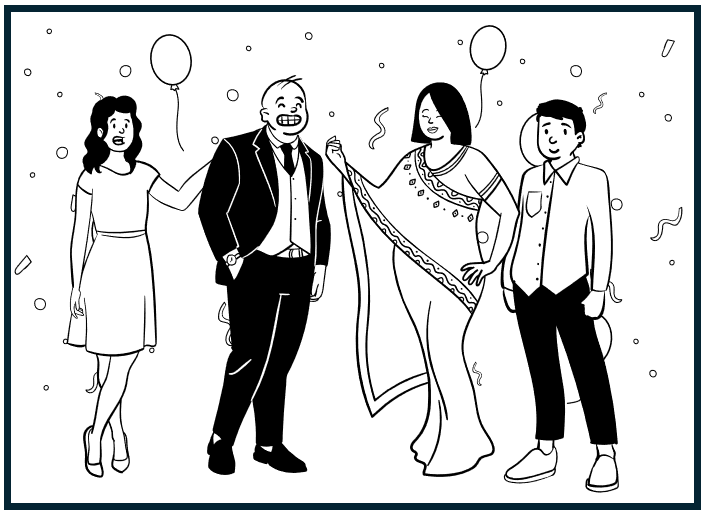


"*You are invited to a wedding of a loved one, and it is an important event for you. Everyone will be dressed up, looking their best. You have a few options for footwear:*

- *Therapeutic shoes given to you by your orthotist that are the best for your foot health but not very stylish*
- *Your own bought daily footwear that are familiar and comfortable but not formal or smart*
- *Stylish shoes perfect for the occasion but they are not the best for your foot health*

*Tell me how you decide on the shoes you wear and why?*"

**Scenario 6: Indoors**


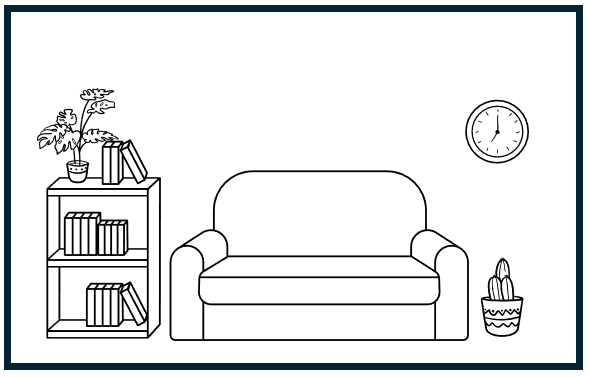


"*You have been out for several hours, and you are tired. You’re ready to relax and take a break.*

*You arrive indoors where you can rest—this could be your home, a friend or family member’s place, or another familiar space where you can relax indoors.*

*Tell me about how your footwear changes, if at all, as you go about your activities indoors.*"

**Final Reflection Questions**

1. Looking back at these scenarios, what was the most important thing that influenced your decisions?
2. Did you find any of the scenarios particularly challenging? Why?
3. Is there anything you wish were different about the footwear options available to you?
